# Transcriptome Analysis Reveals Genes Involved in Thermogenesis in Two Cold-Exposed Sheep Breeds

**DOI:** 10.3390/genes12030375

**Published:** 2021-03-06

**Authors:** Dan Jiao, Kaixi Ji, Hu Liu, Wenqiang Wang, Xiukun Wu, Jianwei Zhou, Yunsheng Zhang, Huitong Zhou, Jon G. H. Hickford, Allan A. Degen, Guo Yang

**Affiliations:** 1 Northwest Institute of Ecological Environment and Resources, Chinese Academy of Sciences, Lanzhou 730070, China; jiaodan@lzb.ac.cn (D.J.); ji_kaixi@163.com (K.J.); 2 University of Chinese Academy of Sciences, Beijing 100049, China; 3 School of Life Sciences, Lanzhou University, Lanzhou 730070, China; Tigerliu18@163.com (H.L.); wangwqcas@163.com (W.W.); zhoujw@lzb.ac.cn (J.Z.); 4 Key Laboratory of Desert and Desertification, Northwest Institute of Eco-Environment and Resources, Chinese Academy of Sciences, Lanzhou 730000, China; wxk163mail@163.com; 5 Key Laboratory of Extreme Environmental Microbial Resources and Engineering, Lanzhou 730000, China; 6 Institute of Animal Husbandry, Xinjiang Academy of Animal Science, Xinjiang 830000, China; xmszys@163.com; 7 Gene-Marker Laboratory, Department of Agricultural Sciences, Lincoln University, Lincoln 7647, New Zealand; Huitong.Zhou@lincoln.ac.nz (H.Z.); Jonathan.Hickford@lincoln.ac.nz (J.G.H.H.); 8 Desert Animal Adaptations and Husbandry, Wyler Department of Dryland Agriculture, Blaustein Institutes for Desert Research, Ben-Gurion University of Negev, Beer Sheva l8410500, Israel; degen@bgu.ac.il

**Keywords:** sheep, cold exposure, transcriptome, adipose tissue

## Abstract

Thermogenesis plays an important role in the survival of sheep exposed to low temperatures; however, little is known about the genetic mechanisms underlying cold adaptation in sheep. We examined 6 Altay (A) and 6 Hu (H) six-month-old ewe lambs. Altay sheep are raised in northern China and are adapted to dry, cold climates, while Hu sheep are raised in southern China and are adapted to warm, humid climates. Each breed was divided into two groups: chronic cold sheep, exposed to −5 °C for 25 days (3 A^c^; 3 H^c^), and thermo-neutral sheep, maintained at 20 °C (3 A^w^; 3 H^w^). The transcriptome profiles of hypothalamus, tail-fat and perirenal fat tissues from these four groups were determined using paired-end sequencing for RNA expression analysis. There are differences in cold tolerance between Hu and Altay sheep. Under cold exposure of the lambs: (1) UCP1-dependent thermogenesis and calcium- and cAMP-signaling pathways were activated; and (2) different fat tissues were activated in Hu and Altay lambs. Several candidate genes involved in thermogenesis including *UCP1*, *ADRB3*, *ADORA2A*, *ATP2A1*, *RYR1* and *IP6K1* were identified. Molecular mechanisms of thermogenesis in the sheep are discussed and new avenues for research are suggested.

## 1. Introduction

As a consequence of the long, harsh winters in the high-altitude alpine meadows in Northern China, sheep often suffer from hypothermia. Consequently, the ability to withstand cold-exposure is vital for their survival. To maintain body temperature when exposed to low air temperature, sheep must increase their heat production (thermogenesis), which involves shivering thermogenesis (ST) and non-shivering thermogenesis (NST) [1]. In new-born lambs, both ST and NST are triggered a few minutes after birth, contributing approximately 46 and 31 percent, respectively, to a neonatal lamb’s maximum metabolism [2]. However, ST can be an inefficient process if it affects the boundary layer of air around the lamb [3], and thus, NST plays a pivotal role in maintaining body temperature during cold-exposure [2].

When lambs, like other mammals, are exposed to cold, the hypothalamic–pituitary axis releases catecholamines to stimulate mitochondria in the adipocytes. These cells generate heat when the β-3 adrenergic receptor (ADRB3) enhances electron transfer chain uncoupling, via uncoupling protein 1 (UCP1). Mammals can also produce heat by triggering creatine cycling [4].

Differences in the capability to withstand cold-exposure in sheep have been reported between breeds [5] and in new-born lambs among lines within a breed in a single flock [6], with the heritability of enhanced resistance to cold-exposure in sheep estimated to be 0.27 [7]. However, to date, little is known about the molecular mechanisms of cold-tolerance in sheep, with most studies focusing on humans and rodents.

In this study, two breeds of sheep were examined. The Altay sheep are indigenous to the Altay Prefecture of Northern Xinjiang Province in China, and are well-adapted to low temperatures. This Prefecture has an average January (winter) temperature of −16.3 °C (Climate-Data.org), with prolonged snow cover. Altay sheep have coarse wool and a fat tail, and are raised for high meat quality [8]. Traditionally, this breed grazes natural pasture all year and does not receive supplementary feed. Hu sheep originated in southern China on the Northern part of the subtropical climate zone, a region that is markedly different climatically from the Altay Prefecture. This breed also has coarse wool and is typically raised in feedlots. It is known for its high prolificacy, rapid early growth and resistance to high humidity and high temperature [9]. Hu sheep were introduced into the central and western regions of China in the 1970s for cross-breeding with local mutton breeds.

Based on different selection backgrounds between breeds, we hypothesized that these breeds would display differences in resistance to cold as well as in genetic regulatory mechanisms when exposed to low temperature. To test this hypothesis, we used an RNA-seq approach to examine the transcriptome profiles of fat and hypothalamus tissues from Altay and Hu sheep when maintained under cold and thermo-neutral conditions.

## 2. Materials and Methods

### 2.1. Animals

Six Altay and six Hu ewe-lambs, six-months of age and of similar body mass (mean 29.2 ± 2.47 kg), were purchased from Zhongmu Sheep Farm in the Altay region in September 2018, where they had been fed alfalfa pellets ad libitum and had free access to water. All lambs had been weaned at 2.5 months of age. Wool length did not differ between the breeds and averaged 8.9 ± 2.15 cm. Each breed was divided into two groups matched for body mass and were maintained in individual metabolic cages in temperature-controlled rooms. For the Altay lambs, one group was cold-exposed at −5 °C (A^c^, *n* = 3) and one group was kept under thermo-neutral conditions at 20 °C (A^w^, *n* = 3). The Hu lambs were divided similarly, one group was cold-exposed (H^c^, *n* = 3) and one group under thermo-neutral conditions at 20 °C (H^w^, *n* = 3). The lambs were acclimatized to the conditions for 7 days prior to the study.

For the cold-exposed lambs, the air temperature was decreased from 20 °C to −5 °C over 10 days (at a reduction of 2.5 °C per day), and then maintained at −5 °C for 25 days. The humidity was 88% ± 6.5. The thermo-neutral lambs were kept at 20 °C for the 35-day duration of the study, and the humidity was 87.5% ± 9.9.

On day 36, rectal temperature of the lambs was measured at 06:00, 14:00 and 22:00 using a mercury thermometer. They were then slaughtered and tissue samples were collected from the hypothalamus, perirenal fat (left kidney) and tail-fat (tail tip). The samples were snap-frozen in liquid nitrogen and stored at −80 °C.

### 2.2. RNA-Seq Analysis—RNA Extraction, Library Construction and Sequencing

A total of 36 RNA samples were extracted from the three different tissues of each lamb in each of the four groups using Trizol reagent (Invitrogen, Carlsbad, CA, USA). The RNA was checked for quality to ensure that the RNA Integrity Number (RIN) was >7.0, and the 28S:18S rRNA ratio was >1.0 and was quantified using an Agilent 2100 bioanalyzer (Thermo Fisher Scientific, MA, USA). The RNA samples from three separate lambs (independent biological replicates: A^c^ (*n* = 3), A^w^ (*n* = 3), H^c^ (*n* = 3) and H^w^ (*n* = 3) for each tissue depot were not pooled.

Poly-A messenger RNA (mRNA) was isolated from the total RNA, which was obtained from the samples, using an oligo dT extraction kit (NEB next poly (A) mRNA magnetic isolation module, NEB, USA). Purified mRNA was fragmented into small pieces with a fragment buffer at an appropriate temperature (Super SCRIPT II Reverse transcriptase, Invitrogen, Carlsbad, CA, USA), and a first-strand cDNA was synthesized from the fragmented mRNA using random oligonucleotide primers and reverse transcriptase (Super SCRIPT II Reverse transcriptase, Invitrogen, Carlsbad, CA, USA). The synthesis of the second-strand used DNA polymerase I and RNase H treatments. A-Tailing Mix and RNA Index Adapters were added by incubating to end repair. The cDNA fragments obtained from the previous step were amplified by PCR, and the products were purified by Ampure XP Beads and then dissolved in EB solution. The product was validated on the Agilent Bioanalyzer (Agilent Technologies 2100, Santa Clara, CA, USA) for quality control. The double stranded PCR products were heated, denatured and circularized by the splint oligo sequence to obtain the final library. The single strand circle DNA (ssCir DNA) was formatted as the final library. The final library was amplified with the ssCir DNA by rolling circle replication (RCR) to enlarge the fluorescent signals at the sequencing process to create DNA nanoball (DNB). The DNBs were loaded into the patterned Nano-array and single end 100 bases reads were generated on a BGIseq500 platform (BGI-Shenzhen, China).

### 2.3. Gene Expression Analysis

The raw sequence reads, which included low quality (reads with more than 20 percent of bases in the total reads had a quality score lower than 15), adaptor (reads that contained the adaptor sequences) and highly unknown base N content (reads which contained more than 5 percent base of undetermined base information), were filtered out using quality control software SOAPnuke (BGI) [10], and then the clean reads were mapped and annotated to the reference genome of *Ovis aries* (Oar_v4.0; https://www.ncbi.nlm.nih.gov/assembly/GCF_000298735.2/ (accessed on 26 April 2019) using the HISAT alignment tool (Center for Computational Biology, Johns Hopkins University, MD, USA). HISAT is based on the burrows-wheeler transform and Ferragina-Manzini (FM) index methods [11]. We mapped clean reads to reference sequence using Bowtie2 for calculating the gene alignment rate [12] (Johns Hopkins University, MD, USA), and then calculated gene expression levels with RSEM (version 1.2.12, University of Wisconsin-Madison, WI, USA), a software package for estimating gene and isoform expression levels from RNA-Seq data [13]. The gene expression levels were normalized by reads per kilobase per million (FPKM) mapped reads. The constrained principal coordinate analysis (cPCoA) was done to visualize classical multidimensional scaling of Bray-Curtis distance matrices by using functions capscale and anova.cca of vegan package in R (version 3.4.1, USA), and the *p* value was calculated by the permutation tests [14,15].

### 2.4. Differentially Expressed Gene Analysis

The hypothalamus, perirenal fat and tail-fat tissues were analyzed to examine the relative level of expression of different transcripts using the DEGseq method in the R statistical software. DEGseq is based on a Poisson distribution, as described by Wang et al. [16]. A heat map showed the marker genes that were generated based on log2 FPKM values using heatmap function in the R statistical software for data analyses and visualization, and a correlation matrix based on Pearson’s correlation coefficient for the marker genes filtered in this study.

Differences in gene expression between the cold and thermo-neutral lambs for the hypothalamus, perirenal fat and tail-fat tissues were tested using pairwise comparisons [16]. The fold changes (FC: FC =$\;\frac{\mathrm{avg}\;\mathrm{FPKM}\;\left(-5\;{}^{\circ}C\right)}{\mathrm{avg}\;\mathrm{FPKM}\;\left(20\;{}^{\circ}C\right)}$) were estimated according to the normalized gene expression level in each sample. A threshold of adjusted *p* value ≤ 0.01 and absolute value of |log2 FC| ≥ 1 were used to determine significant differences in gene expression among the four groups. The adjusted *p*-value was calculated using the Benjamini and Hochberg False Discovery Rate concept [17].

### 2.5. Function Enrichment and Analyses

Gene ontology (GO) enrichment analysis provides a systematic method of defining the function of gene products [16,18], and includes GO functional analysis and GO enrichment analysis. GO functional analysis aligns DEGs sequence to GO database (http://www.geneontology.org/ (accessed on 26 April 2019) for functional annotation. Based on the GO annotation results, GO enrichment analysis uses the phyper function in the R statistical software to calculate hypergeometric probabilities of DEGs (version 3.4.1, Lucent Technologies, AZ, USA).

The DEGs were enriched on three default GO terms, ‘molecular function’, ‘biological process’ and ‘cellular component’. A level of adjusted *p* ≤ 0.01 was used to accept whether DEG results from GO analyses were significant.

The KEGG pathway enrichment was used to identify genes and metabolic pathways that involved the DEGs, according to methods described in the KEGG database [19] (http://www.kegg.jp/kegg/pathway.html/ (accessed on 26 April 2019). The KEGG enrichment analysis method was consistent with the GO enrichment.

### 2.6. RNA-Seq Validation by Quantitative Real-Time PCR

To test selected gene expression differences which had been identified by the RNA-seq analysis, the expressions of four candidate genes, namely, *UCP1*, *RYR1* (ryanodine receptor 1), *ADIPOQ* and *LPL* (lipoprotein lipase) were analyzed using an RT-qPCR approach. The primers for RT-qPCR validation were designed using Oligo7 (Wojciech Rychlik, USA) (Table 1), with *β-actin* as a reference gene to verify the relative level of expression. The RT-qPCR amplification of cDNA pools used a PrimerScript RT reagent kit (Takara) with gDNA Erase (Takara), according to the manufacturer’s instructions. The RT-qPCR reactions used an Agilent Mx3000P system (Mx3000P, Stratagene, Agilent, Santa Clara, CA, USA), and reactions contained 2 µL of cDNA, 0.8 µL forward and reverse primers (10 uM), 10 µL TB Green^TM^ Premix Ex *Taq* II, 6 µL RNAase free water, and 0.4 µL ROX Reference Dye II (50×) in a total volume of 20 µL. The thermal profile for amplification followed a two-step approach: 1) pre-denaturation for 15 s at 95 °C; then 2) 5 s at 95 °C and 34 s at the Tm listed in Table 1 for 40 cycles. Changes in gene expressions were determined by the 2^−^^△△ct^ method [20].

### 2.7. Protein-Protein Interaction Network Analyses

All the differentially expressed transcripts used a hierarchical clustering algorithm and prediction methods were based on information derived from string database (https://string-db.org (accessed on 26 April 2019). The PPI pairs of proteins encoded by DEGs were retrieved using the tools of Cytoscape (version 3.6.1) [21]. The candidate genes (*UCP1*, *ATP2A1*, *RYR1* and *IP6K1*) were chosen to construct a regulatory network.

### 2.8. Statistical Analyses

All qPCR and rectal temperature data were analyzed by t-tests and one-way ANOVA. (IBM SPSS statistics 20.0 for Windows, SPSS Inc., Chicago, IL, USA). A level of *p* < 0.05 was accepted as significant. Results are presented as means ± SEM.

### 2.9. Availability of Data and Materials

The datasets generated and analyzed during the current study are available confidentially to editors and reviewers, and all transcriptome data were submitted to the NCBI sequence read archive (SRA: accession PRJNA542078).

### 2.10. Ethics Approval and Consent to Participate

The study design and all procedures on sheep were approved by the Academic Committee of Northwestern Institute of Eco-Environment Resources, Chinese Academy of Sciences (protocol number: CAS201810082).

## 3. Results

**Figure 1:**
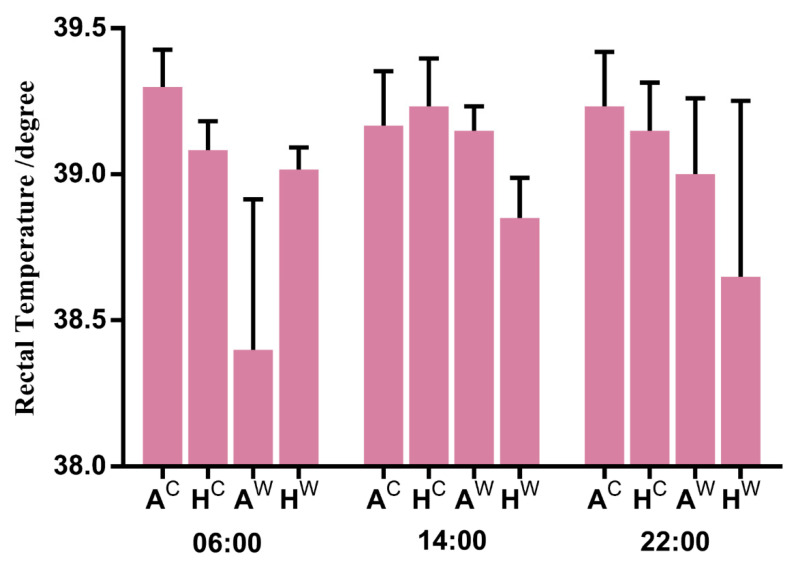
Rectal temperature of Altay and Hu lambs maintained at different air temperatures. The rectal temperatures of the lambs were measured on day 36 of the study at 06:00, 14:00 and 22:00. A^c^: Altay lambs at −5 °C, H^c^: Hu lambs at −5 °C, A^w^: Altay lambs at 20 °C, and H^w^: Hu lambs at 20 °C. Values are means ± SD from 6 lambs per group.

Rectal temperatures ranged between 38.4 °C and 39.3 °C. All lambs maintained normal rectal temperature (Figure 1) and appeared to be in good health throughout the study.

### 3.1. Sequencing and Mapping

**Figure 2:**
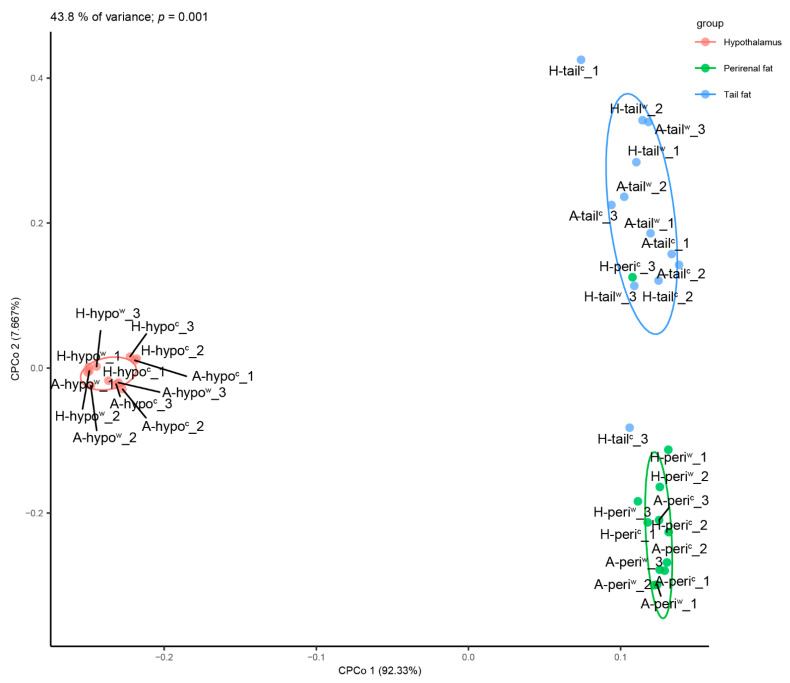
Constrained PCoA plot of Bray–Curtis distances constrained by global gene FPKM (48.3% of variance explained, *p* = 0.001; *n* = 36). Each point corresponds to a different sample, and each color represents a type of tissue. The percentage of variation indicated in each axis corresponds to the percentage of the total variance explained by the projection. A-hypo^c^: hypothalamus tissue of −5 °C Altay lambs; H-hypo^c^: hypothalamus tissue of −5 °C Altay lambs; A-hypo^w^: hypothalamus tissue of 20 °C Altay lambs; H-hypo^w^: hypothalamus tissue of 20 °C Hu lambs. A-peri^c^: perirenal fat tissue of −5 °C Altay lambs; H-peri^c^: perirenal fat tissue of −5 °C Altay lambs; A-peri^w^: perirenal fat tissue of 20 °C Altay; H-peri^w^: perirenal fat tissue of 20 °C Hu lambs. A-tail^w^: tail-fat tissue of −5 °C Altay lambs; H-tail^c^: tail-fat tissue of −5 °C Altay lambs; A-tail^w^: tail-fat tissue of 20 °C Altay lambs; H-tail^w^: tail-fat tissue of 20 °C Hu lambs.

To examine the global difference between different tissues, breeds and treatments in the transcriptome sequence in 36 samples, constrained principal coordinate analysis (CPCoA) by Bray-Curtis distances was done for every biological repeat of different tissue and treatment (Figure 2). The 36 samples could explain 48.3% of variance of the total and the different tissues were clustered well (*p* = 0.001). The hypothalamus and adipose tissue were distinguished significantly in the CPCo 1 (92.3% of the variance of the total explanation value of 48.3%), and tail fat and perirenal fat were also distinguished in the CPCo 2 (7.7% of variance in the CPCo 1 of 48.3%). The samples of different tissues were divided into three groups. There was more organizational distance between adipose tissues than between breeds or temperature treatment. The distance between duplicates was relatively far, which meant dispersion of the sample was relatively large. In addition, the distance between three biological duplicates in the fat tissue of cold exposed Hu lambs was relatively far when compared to the other treatments. The PCoA indicated that the dispersion in the H-tail^c^ and H-peri^c^ was caused by individual differences among the three cold-exposed Hu lambs, but this dispersion did not affect subsequent analyses.

The average matching ratio of the clean reads to the genome was 87.8%, and the average matching ratio of the clean reads to the gene sequence was 78.2%. The sequencing and mapping data are summarized in Supplementary File S2: Table S1.

### 3.2. Gene Annotation

**Figure 3:**
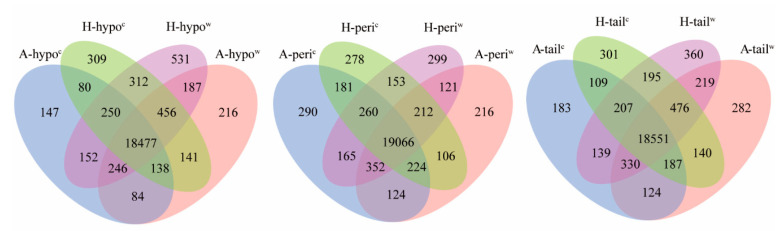
Venn diagrams representing annotated genes in hypothalamus tissue, tail-fat tissue and perirenal fat tissue of cold-exposed Altay and Hu lambs; A-hypo^c^: hypothalamus tissue of −5 °C Altay lambs; H-hypo^c^: hypothalamus tissue of −5 °C Altay lambs; A-hypo^w^: hypothalamus tissue of 20 °C Altay lambs; H-hypo^w^: hypothalamus tissue of 20 °C Hu lambs. A-peri^c^: perirenal fat tissue of −5 °C Altay lambs; H-peri^c^: perirenal fat tissue of −5 °C Altay lambs; A-peri^w^: perirenal fat tissue of 20 °C Altay; H-peri^w^: perirenal fat tissue of 20 °C Hu lambs. A-tail^w^: tail-fat tissue of −5 °C Altay lambs; H-tail^c^: tail-fat tissue of −5 °C Altay lambs; A-tail^w^: tail-fat tissue of 20 °C Altay lambs; H-tail^w^: tail-fat tissue of 20 °C Hu lambs.

A total of 23,736 genes were annotated according to the Oar reference genome Oar_v4.0, including 20,649 previously identified genes and 3087 potentially novel genes, that is, where genes mapped to unannotated regions of the genome. Venn diagrams compared the different tissues, temperatures and breeds according to the global genes which were annotated (Figure 3). In a comparison between breeds, the number of different annotated genes in the hypothalamus and tail-fat tissues in the cold-exposed group were greater than in other compared groups.

### 3.3. Analysis of the DEGs

**Figure 4:**
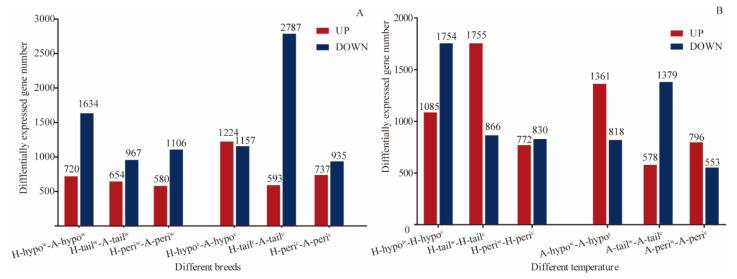
The expression of DEGs in hypothalamus tissue, tail-fat tissue and perirenal fat tissue of cold-exposed Altay and Hu lambs. (**A**) DEGs expressed in Altay and Hu lambs at same air temperature and tissues. (**B**) DEGs expressed at different air temperature (−5 °C and 20 °C) within the same breed and tissues. All the compared groups used Hu and thermo-neutral Altay lambs as control groups. H-hypo^w^-A-hypo^w^: hypothalamus tissue of the 20 °C Altay lambs compared to the 20 °C Hu lambs. H-tail^w^-A-tail^w^: tail-fat tissue of the 20 °C Altay lambs compared to the 20 °C Hu lambs. H-peri^w^-A-peri^w^: perirenal fat tissue of the 20 °C Altay lambs compared to the 20 °C Hu lambs. H-hypo^c^- A-hypo^c^: hypothalamus tissue of the −5 °C Altay lambs compared to the −5 °C Hu lambs; H-tail^c^-A-tail^c^: tail-fat tissue of the −5 °C Altay lambs compared to the −5 °C Hu lambs. H-peri^c^-A-peri^w^: perirenal fat tissue of the −5 °C Altay lambs compared to the −5 °C Hu lambs. H-hypo^w^-H-hypo^c^: hypothalamus tissue of the −5 °C Hu lambs compared the 20 °C Hu lambs. H-tail^w^-H-tail^c^: tail-fat tissue of the −5 °C Hu lambs compared the 20 °C Hu lambs. H-peri^w^-H-peri^c^: perirenal fat tissue of the −5 °C Hu lambs compared the 20 °C Hu lambs. A-hypo^w^-A-hypo^c^: hypothalamus tissue of the −5 °C Altay lambs compared the 20 °C Hu lambs. A-tail^w^-A-tail^c^: tail-fat tissue of the −5 °C Altay lambs compared the 20 °C Hu lambs. A-peri^w^-A-peri^c^: perirenal fat tissue of the −5 °C Altay lambs compared the 20 °C Hu lambs.

Comparisons of the DEGs up- and down regulation between the two breeds and at the different temperatures for the three tissues are presented in Figure 4. More DEGs were down-regulated in the hypothalamus, tail-fat and perirenal fat tissues in the 20 °C Altay lambs when compared to the 20 °C Hu lambs. After exposure to low temperature, more DEGs were down-regulated in the hypothalamus of Hu lambs and tail-fat of Altay lambs, and up-regulated in the hypothalamus and perirenal fat tissue of the Altay lambs and the tail-fat tissue of the Hu lambs.

**Figure 5:**
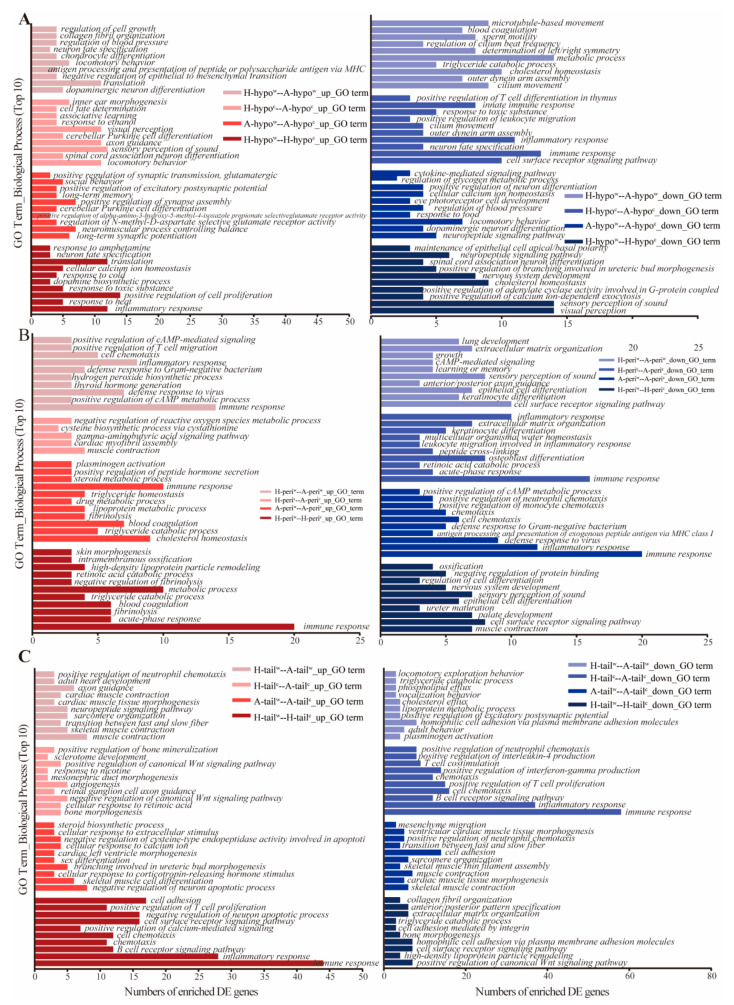
Top 10 up- and down-regulated DEGs enriched GO terms in different tissues and sheep breeds under cold exposure. (**A**): GO terms enriched in the hypothalamus, (**B**): GO terms enriched in the perirenal fat tissue, (**C**): GO terms enriched in the tail-fat tissue. H-hypow- A-hypow: hypothalamus tissue of the 20 °C Altay lambs compared to the 20 °C Hu lambs. H-hypoc- A-hypoc: hypothalamus tissue of the −5 °C Altay lambs compared to the −5 °C Hu lambs. H-hypow-H-hypoc: hypothalamus tissue of the −5 °C Hu lambs compared to the 20 °C Hu lambs. A-hypow-A-hypoc: hypothalamus tissue of the −5 °C Altay lambs compared to the 20 °C Hu lambs. H-tailw-A-tailw: tail-fat tissue of the 20 °C Altay lambs compared to the 20 °C Hu lambs. H-tailc-A-tailc: tail-fat tissue of the −5 °C Altay lambs compared to the −5 °C Hu lambs. H-tailw-H-tailc: tail-fat tissue of the −5 °C Hu lambs compared to the 20 °C Hu lambs. A-tailw-A-tailc: tail-fat tissue of the −5 °C Altay lambs compared to the 20 °C Hu lambs. H-periw-H-peric: perirenal fat tissue of the −5 °C Hu lambs compared to the 20 °C Hu lambs. H-periw-A-periw: perirenal fat tissue of the 20 °C Altay lambs compared to the 20 °C Hu lambs. H-peric-A-periw: perirenal fat tissue of the −5 °C Altay lambs compared to the −5 °C Hu lambs. A-periw-A-peric: perirenal fat tissue of the −5 °C Altay lambs compared to the 20 °C Hu lambs.

The GO (gene ontology) analyses revealed that the DEGs could be categorized into molecular function (MF), cellular component (CC) and biological process (BP). The selected significant GO term annotations are presented in Supplementary File S4: Table S3 (adjusted *p* value < 0.01). For the MF GO category, DEGs in the hypothalamus tissue were enriched in transporter activity, heme and tetrapyrrole binding and ion transmembrane transporter channel activity related functions; the perirenal fat tissue was enriched in receptor activity and heme binding; and the tail-fat tissue was enriched in chemokine, cytokine activity, receptor binding and receptor activity. For the CC GO category, DEGs in the hypothalamus tissue were enriched in the plasma membrane parts and both intrinsic and integral components of the membrane; the perirenal fat tissue was enriched in membrane parts and extracellular regions; and the tail-fat tissue was enriched in intrinsic and integral components of the membrane, and membrane parts. For the top 10 up- and down-DEGs enriched CC and MF terms (q < 0.05), DEGs in the hypothalamus, perirenal fat and tail-fat tissues are presented in Supplementary File S1: Figure S1 and Figure 5. The BP term related to heat production was up-regulated in the hypothalamus of the −5 °C compared to 20 °C Hu lambs. The MF term of ATP binding was up-regulated in the perirenal fat tissue of the −5 °C Altay lambs compared to −5 °C Hu lambs and was up-regulated in the tail-fat of the −5 °C compared to 20 °C Hu lambs. A large number of up-regulated DEGs were enriched in the GO terms of perirenal fat and hypothalamus tissues of the −5 °C Altay lambs and tail-fat tissue of the −5 °C Hu lambs.

A summary of the KEGG (Kyoto Encyclopedia of Genes and Genomes) pathway analyses of the DEGs in the different tissues and groups according to q-value (q < 0.01) is presented in Supplementary File S5: Table S4. We identified the most significantly differentially expressed pathways (top 10) in each tissue in the up- and down-regulated probes separately (Supplementary File S6: Table S5).

In the hypothalamus, the calcium signaling pathway was significantly enriched in the breeds during cold exposure. The phosphatidylinositol signaling system and cyclic AMP (cAMP) signaling pathway were both enriched in the −5 °C Altay and Hu lambs, but more DEGs were enriched in the Hu lambs. Both the Rap1- and Ras signaling pathways were significantly enriched in the Hu lambs, but not in the Altay lambs. cAMP- and calcium signaling pathways were up-regulated in the −5 °C Altay lambs compared to the 20 °C Altay lambs and −5 °C Altay lambs compared to the −5 °C Hu lambs, but down-regulated in the −5 °C Hu lambs compared to the 20 °C Hu lambs. In the perirenal fat tissue, the NF-kappa B-, the calcium- and the rap1-signaling pathways were significantly enriched in the 20 °C Altay lambs compared to the 20 °C Hu lambs and the −5 °C Altay lambs compared to the −5 °C Hu lambs. With exposure to low temperature, the Ras-signaling pathway and steroid hormone biosynthesis, and cAMP signaling pathway were significantly enriched in the Altay and Hu lambs. Fat digestion and absorption pathway was up-regulated in the −5 °C Altay lambs compared to the 20 °C Altay lambs. The cAMP signaling pathway, ABC transporters, adrenergic signaling in cardiomyocytes, carbohydrate digestion and absorption and cardiac muscle contraction were all up-regulated in the −5 °C Altay lambs compared to the −5 °C Hu lambs. ABC transporters were down-regulated in the −5 °C Hu lambs compared to the 20 °C Hu lambs. In the tail-fat tissue, the two breeds were enriched in the calcium-, phosphatidylinositol-, cAMP-, Rap1- and Ras signaling pathways at low temperature, but fewer DEGs were enriched in the −5 °C Altay lambs in the calcium signaling pathway than in the Hu lambs. The PI3K-Akt signaling pathway and adrenergic signaling in cardiomyocytes were down-regulated in the −5 °C Altay lambs compared to the 20 °C Altay lambs. The NF-kappa B-, rap1- and MAPK signaling pathways and cytokine-cytokine receptor interaction were up-regulated in the −5 °C Hu lambs compared to the 20 °C Hu lambs, and calcium signaling pathway, insulin secretion, ABC transporters, ascorbate and aldarate metabolism and pentose and glucoronate interconversions were down-regulated in the −5 °C Hu lambs compared to the 20 °C Altay lambs. In contrast, pathways of ascorbate and aldarate metabolism and pentose and glucoronate interconversions were up-regulated in the −5 °C Altay lambs compared to the −5 °C Hu lambs, and NF-kappa B-, rap1-, ras-, MAPK- and PI3K-Akt signaling pathways, adrenergic signaling in cardiomyocytes pathway and cardiac muscle contraction pathways were all down-regulated in the −5 °C Altay lambs compared to the −5 °C Hu lambs.

### 3.4. Validation of RNA-Seq Results by RT-qPCR

To substantiate the RNA-seq results, four DEGs from different pathways, including *UCP1*, *RYR1*, *ADIPOQ* and *LPL*, were selected for RT-qPCR analysis. The *UCP1* and *ADIPOQ* genes were involved in the UCP1-dependent thermogenesis pathway [22], *LPL* gene was not only involved in the UCP1-dependent thermogenesis of brown adipose tissue (BAT) but also in the thermogenesis of hypothalamus [23,24], and *RYR1* gene participated in the calcium signaling pathway [25]. The qPCR results were in agreement with RNA-seq results in expression trends, so the transcriptome results were reliable and could be used for subsequent analysis. The relative quantities of these genes from different tissues in all four lamb groups are shown in Supplementary File S1: Figure S2.

### 3.5. Protein-Protein Interaction (PPI) Analyses

**Figure 6:**
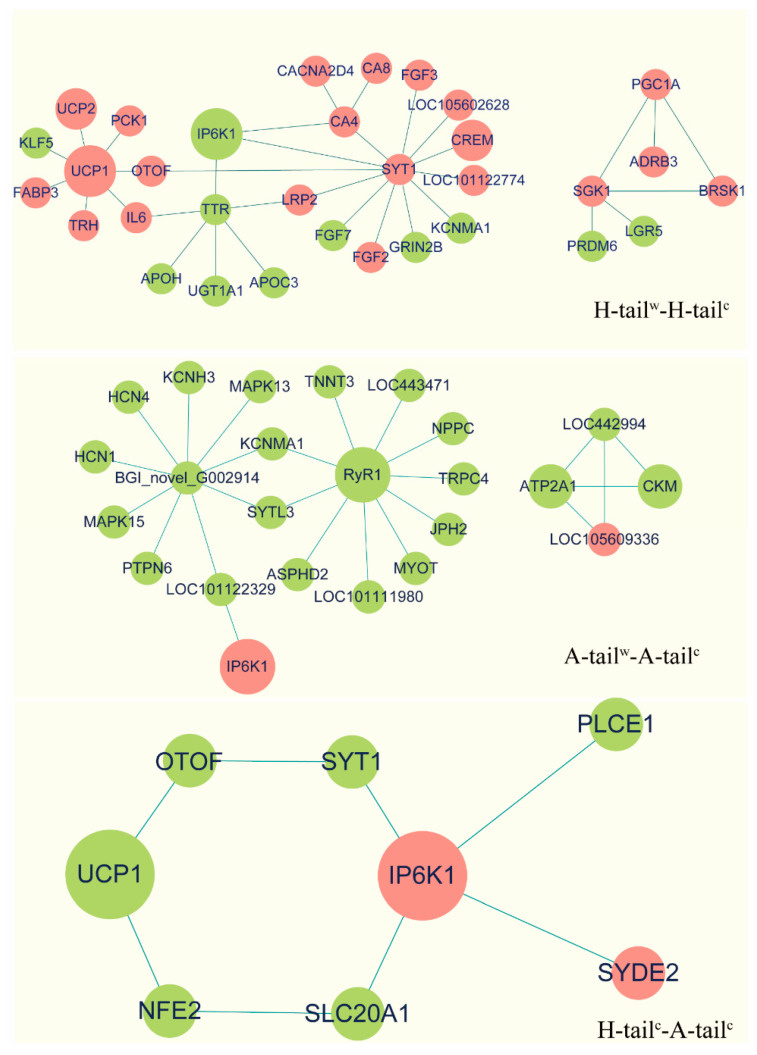
The interaction of proteins between different groups in the tail-fat of cold-exposed Altay and Hu lambs. Red circles represent up-regulation of genes, green circles represent down-regulation of genes. A-tail^c^: tail-fat tissue of −5 °C Altay lambs; H-tail^c^: tail-fat tissue of −5 °C Altay lambs; A-tail^w^: tail-fat tissue of 20 °C Altay lambs; H-tail^w^: tail-fat tissue of 20 °C Hu lambs.

The PPI analysis, done on Cytoscape software, was based on transcriptome DEGs and String database, which evaluates and scores the relationship between two genes. Protein clustering analyses are illustrated in Figure 6. The IP6K1 protein had an indirect relationship with the RYR1 protein and UCP1 gene in the tail-fat tissue of −5 °C Altay lambs compared to the 20 °C Altay lambs and the −5 °C Hu lambs compared to the 20 °C Hu lambs. The IP6K1 protein had a direct relationship with the UCP1 protein in the tail-fat tissue of −5 °C Altay lambs compared to the −5 °C Hu lambs. (A direct relationship indicates that two DEGs can be linked by their common DEGs between control and treatment groups, and there is a correlation between them in gene function. An indirect relationship indicates that two DEGs have no common DEGs between control and treatment groups). The genes *ATP2A1* (*SERCA1*) and *CKM* (creatine kinase) had a direct protein-protein interaction with each other in the tail-fat tissue of the −5 °C Altay lambs compared to the 20 °C Altay lambs, but there was no relationship in the −5 °C tail-fat of Hu lambs. The UCP1 protein did not have a relationship in the tail-fat tissue of −5 °C Altay lambs compared to the 20 °C Altay lambs, which suggests there may be different strategies of thermogenesis in the two breeds.

### 3.6. Candidate Genes Analyses

**Figure 7:**
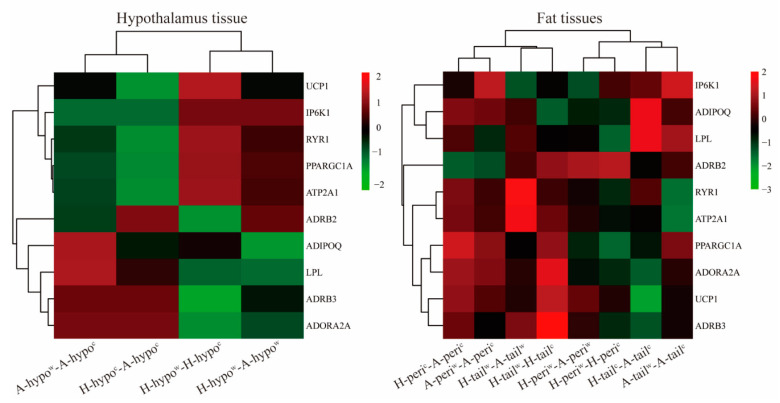
The heat map of candidate gene clustering in the hypothalamus and fat tissues. H-hypo^w^- A-hypo^w^: hypothalamus tissue of the 20 °C Altay lambs compared to the 20 °C Hu lambs. H-hypo^c^- A-hypo^c^: hypothalamus tissue of the −5 °C Altay lambs compared to the −5 °C Hu lambs. H-hypo^w^-H-hypo^c^: hypothalamus tissue of the −5 °C Hu lambs compared to the 20 °C Hu lambs. A-hypo^w^-A-hypo^c^: hypothalamus tissue of the −5 °C Altay lambs compared to the 20 °C Hu lambs. H-tail^w^-A-tail^w^: tail-fat tissue of the 20 °C Altay lambs compared to the 20 °C Hu lambs. H-tail^c^-A-tail^c^: tail-fat tissue of the −5 °C Altay lambs compared to the −5 °C Hu lambs. H-tail^w^-H-tail^c^: tail-fat tissue of the −5 °C Hu lambs compared to the 20 °C Hu lambs. A-tail^w^-A-tail^c^: tail-fat tissue of the −5 °C Altay lambs compared to the 20 °C Hu lambs. H-peri^w^-H-peri^c^: perirenal fat tissue of the −5 °C Hu lambs compared to the 20 °C Hu lambs. H-peri^w^-A-peri^w^: perirenal fat tissue of the 20 °C Altay lambs compared to the 20 °C Hu lambs. H-peri^c^-A-peri^w^: perirenal fat tissue of the −5 °C Altay lambs compared to the −5 °C Hu lambs. A-peri^w^-A-peri^c^: perirenal fat tissue of the −5 °C Altay lambs compared to the 20 °C Hu lambs. Candidate genes were clustered with log2 changes (log2 FC >1, *p* < 0.05) in all compared groups included in this heatmap. Hierarchical clustering of samples was based on Pearson’s correlation coefficient, and scales were performed in rows. FC, fold change.

The heat map of several candidate genes between breeds and tissues are presented in Figure 7. These candidate genes were selected according to results of GO and KEGG enriched analysis and PPI analysis between different compared groups. The heatmap was clustered by different compared groups with thermogenic related candidate genes, which showed tissue and breed differences after cold exposure. These candidate genes in the cold-exposed Hu and Altay lambs had opposite regulatory trends. In the hypothalamus tissue, there was a large difference between Altay and Hu lambs after cold exposure. In the fat tissues, the tail-fat and perirenal fat of cold exposed Hu and Altay lambs were clustered closely. The detailed list of DEGs is presented in Supplementary File S3: Table S2.

## 4. Discussion

Transcriptome profiling provides new insights into the mechanisms underlying the tolerance of sheep to cold-exposure. In this context, we undertook a comparative RNA-Seq study of three tissues (hypothalamus, tail-fat and perirenal fat tissues) from cold-exposed Altay and Hu sheep. All lambs maintained normal rectal temperatures with no significant difference among groups. The highest number of DEGs occurred in the tail-fat of −5 °C Altay and Hu lambs (3380 in total) with 2787 down-regulated DEGs and 593 up-regulated DEGs, which suggests that some genes expressed in the tail-fat tissue have important roles in response to cold when compared to those in other tissues. The Altay sheep have a fat tail which stores energy and is important in overcoming periods of sparse winter pasture, and also in preventing hypothermia [26,27]. In contrast, Hu sheep have a short fat tail [28], and this may explain, at least in part, the substantial difference in tail fat between Hu and Altay sheep after cold exposure.

Thermogenic genes like *UCP1* were up-regulated in tail-fat of −5 °C Hu lambs compared to the 20 °C Hu lambs, in perirenal fat of −5 °C Altay lambs compared to the −5 °C Hu lambs and −5 °C Altay lambs compared to the 20 °C Altay lambs. The results indicated that adipose tissue was browning and thermogenic responses had occurred in tail-fat of Hu lambs and in perirenal fat of Altay lambs under cold exposure.

The GO analysis revealed that more up-regulated DEGs enriched in a larger number of metabolic process-related GO terms in Hu lambs than in Altay lambs after cold exposure, which suggests that Hu lambs had a greater response to low temperature. Up-regulated DEGs were significantly enriched in the GO term of GTP binding in tail fat of −5 °C compared to 20 °C Hu lambs, and up-regulated DEGs were significantly enriched in the GO term of ATP binding in perirenal fat of −5 °C Altay lambs compared to −5 °C Hu lambs. GTP binding capacity to BAT mitochondria or UCP1 determines the thermogenesis potential of BAT to respond to cold exposure [29]. At thermoneutrality, the mitochondria are coupled since UCP1 is largely inhibited by endogenous ATP. Upon acute cold stimulation, fatty acids (FAs) are released which can both activate UCP1 and deplete endogenous ATP by FA activation where ATP is converted to AMP [30,31]. The results indicated that Altay and Hu lambs activated different fat tissues for thermogenesis under cold exposure. In our previous study [32], feed intake was greater but feed conversion efficiency was lesser in Hu than in Altay lambs at low temperature. Average daily gain was significantly increased in both breeds at −5 °C, but the average daily gain of Altay lambs was higher than that of Hu lambs (Supplementary Table S5). This suggested that Hu lambs allotted more energy into maintaining body temperature than into growth compared to Altay lambs under low air temperature. The concentrations of blood glucose and non-esterified fatty acids (NEFA) were greater in Hu than in Altay sheep under thermo-neutral conditions [32] but did not differ between breeds when exposed to cold [33]. These results indicate that cold exposure induced responses from different fat tissues in different breeds for thermogenesis, and that Altay lambs are more resistant to cold than Hu lambs.

### 4.1. Pathways Identified in Low Temperature Challenges—cAMP and Calcium Signaling Pathways

According to KEGG pathway analyses, upon cold exposure, Hu lambs activated more signal transduction pathways in the hypothalamus and tail-fat tissue to regulate thermogenesis than Altay lambs. In contrast, the Altay lambs activated more pathways in the perirenal fat tissue at low temperatures. The results revealed that the cAMP- and calcium signaling pathways were the major pathways involved in the different tissues from the −5 °C Altay and Hu lambs. The cAMP signaling pathway was enriched in all cold-exposed lambs, which suggested that it plays an important role in the response to cold exposure for different sheep breeds. In addition, cAMP was also enriched in the tail-fat of the −5 °C compared to 20 °C Hu lambs and in the hypothalamus in all cold-exposed lambs. cAMP, known as the second messenger, is involved in triggering energy metabolic and thermogenic processes. Low temperature stimulates an increase in the intracellular concentration of cAMP [34,35,36], which activates transcription of thermogenic-related genes by a series of phosphorylation reactions [37,38]. The results suggested that the hypothalamus was involved in the response to cold exposure in Hu and Altay lambs, and tail-fat of Hu lambs was involved through the cAMP signaling pathway. Furthermore, cAMP also modulates the Ca^2+^ transport pathway [39], leading to an increase in heat production [40]. Although the calcium signaling pathway was enriched in all groups (except for tail-fat of the 20 °C Altay compared to Hu lambs) (Supplementary Table S4), further analysis found that more down-regulated DEGs enriched in the hypothalamus and tail-fat of −5 °C compared to 20 °C Hu lambs, and more up-regulated DEGs enriched in the hypothalamus of −5 °C Altay lambs (Supplementary Table S5). The calcium signaling pathway was another enriched pathway in the present study. It was reported to be activated in humans in both the sarcoplasmic and endoplasmic reticulum (ER) and mitochondria [41,42], as well as in pre-adipocytes [43], with a close calcium-dependent functional association between the ER and mitochondria [41,42]. Calcium ions can be transferred from the ER to the mitochondrial matrix via the calcium signaling pathway, which leads to activation of metabolic functions of the mitochondria in skeletal muscles [44,45]. In addition, it was reported that an increase of cytosolic Ca^2+^ concentration from a basal level of 0.05 uM to 0.2–0.7 uM, could trigger heat production in BAT cells in rabbits [46]. The results suggested that the calcium signaling pathway participated in hypothalamic thermogenesis of Altay sheep. We reasoned that the enrichment of down-regulated DEGs in the calcium signaling pathway might be due to the UCP1-dependent thermogenesis in the tail fat of cold exposed Hu lamb and that the heat generation mode of the calcium signaling pathway was restricted.

There was evidence that a calcium signaling pathway was enriched in the present study, although no direct *UCP1* thermogenesis pathway was identified. Some marker genes like *UCP1*, *UCP2* (uncoupling protein 2) and *ADRB3* gene were up-regulated to a greater extent in perirenal fat of the −5 °C Altay lambs compared to 20 °C Altay lambs, and in tail-fat of the −5 °C Hu lambs compared to 20 °C Hu lambs. These results indicated that the tail-fat and perirenal fat of Hu and Altay lambs were involved in *UCP1* thermogenesis.

Given the different homeostatic pathways and genes identified in this study, we concluded that all three tissues were involved in producing heat; but under a cold challenge, the cAMP signaling pathway displayed significant differences among breeds. The calcium signaling pathway may be inhibited in producing heat, being dependent on *UCP1* in Hu lambs. The hypothalamus of Altay and Hu lambs participated in heat production through different pathways, and different fat tissues of Hu and Altay lambs were involved in UCP1 thermogenesis. The candidate genes heatmap analysis indicated that the pathways related candidate genes had opposite regulatory effects between breed and within the same breed after cold exposure in hypothalamus tissue, but not in fat tissues. The large difference in the hypothalamus tissue suggests that it may play an important role in the cold response in sheep.

### 4.2. Analysis of the Genes Participating in Calcium and cAMP Signaling Pathways

We screened the DEGs related to thermogenesis from the significant enriched signal transduction pathways within the GO categories.

In the present study, adenosine A2A receptor (*ADORA2A*) was an important gene in the cAMP signaling pathway, which was down-regulated in the perirenal fat and tail-fat, and had a down-regulated trend in the hypothalamus of −5 °C Altay lambs compared to Hu lambs. It was up-regulated in perirenal fat of −5 °C compared to 20 °C Altay lambs and tail-fat of −5 °C compared to 20 °C Hu lambs. *ADORA2A*, one of the receptors of adenosine in this study, is an important thermogenic marker expressed in adipose tissues [47] that could increase human and rodent lipolysis by inducing noradrenaline (NE) in BAT. Our results suggest that under cold exposure, *ADORA2A* gene regulated lipolysis in perirenal fat of Altay lambs and tail-fat of Hu lambs.

Another important receptor of adenosine, *ADRB3* gene, was up-regulated in the tail-fat of 20 °C Altay lambs compared to Hu lambs, which indicated there was a breed difference in this gene. *ADRB3* was also down-regulated in the hypothalamus and tail-fat of the −5 °C Altay lambs compared to Hu lambs, and up-regulated in the tail-fat of −5 °C compared to 20 °C Hu lambs. The ADRBs are normally expressed on the surface of brown adipocytes in BAT and they mediate mitochondrial biogenesis and thermogenesis [48,49,50]. In the study of hibernating mammals, the *ADRB3* gene was expressed greater at the end of the hibernation period [51]. Studies have also confirmed that *ADRB3* is important in hypothalamic–pituitary–adrenal regulatory activities [52,53]. These results suggest a breed difference in the expressions of *ADRB3* in tail-fat, which may be due to the different tail types between breeds. Up-regulated in tail-fat of Hu lambs after cold exposure indicated that the *ADRB3* gene participated in heat production of tail-fat in the −5 °C Hu lambs.

In the present study, a large number of calcium signaling related genes were correlated with cold tolerance. *ATP2A1* and *RYR1* were up-regulated in tail-fat of the −5 °C Altay lambs. Sarcoplasmic and ER Ca^2+^-ATPase 1 are expressed on vesicles derived from BAT endoplasmic reticulum, and is bound to the inner membrane of BAT mitochondria [54]. In BAT mitochondria, *ATP2A1* can generate heat when Ca^2+^ concentrations are similar to the intracellular Ca^2+^ concentrations during adrenergic stimulation [55]. Studies on *RYR1* in mammals have focused on thermogenesis in skeletal muscles [56,57,58]. Research on hibernating mammals reported that muscle-derived *ATP2A1* and *RYR1* were regulated by the Ca^2+^ pump, and decreased during torpor but increased upon arousal from hibernating [25]. We presumed that both *ATP2A1* and *RYR1* relied on the calcium signaling pathway involved in heat production of cold exposed Altay lambs in our study. The 5-hydroxytryptamine receptor 2B (*HTR2B*) plays an important role in lipolysis in WAT by activating hormone sensitive lipase (*LIPE*), and it could alter Ca^2+^ influx and the rate of mitochondrial oxidative consumption [59,60,61]. *HTR2B* and *LIPE* were up-regulated in the hypothalamus and perirenal fat tissue of the −5 °C Hu lambs. These genes promote fat mobilization and heat production in cold-exposed lambs, when the calcium- and cAMP signaling pathways enhance heat production in cold-exposed sheep.

### 4.3. Analysis of the Genes Participating in the UCP1 Related Thermogenesis Pathway

*UCP1*, formerly known as “thermogenin”, is a key thermogenic gene in cold-induced NST [62] and one of the markers of adipose tissue browning. Up-regulation of the *UCP1* gene in perirenal fat of −5 °C Altay lambs and in tail-fat of the −5 °C Hu lambs indicated that these adipose tissues underwent browning. Its transcription factor *PPARGC1A* (peroxisome proliferator-activated receptor γ coactivator-1 α), which regulates mitochondrial biogenesis and respiration, adaptive thermogenesis, gluconeogenesis and other metabolic processes [63], was up-regulated in tail-fat of −5 °C Altay and Hu lambs, and perirenal fat of −5 °C Altay lambs. The up-regulation of two key genes in the *UCP1* thermogenic pathway verified that the traditional thermogenic pathway existed in this study. The up-regulation of these three key UCP1-dependent thermogenic genes (including *ADRB3*) in perirenal fat of −5 °C Altay lambs and tail-fat of the −5 °C Hu lambs verified that cold exposure induced UCP1-dependent thermogenesis in different adipose tissue of different sheep breeds. The *PPARGC1A* and *UCP1* genes were inconsistently expressed in tail-fat of cold-exposed Altay lambs because *PPARGC1A* gene was an upstream gene, which might not involve transcriptional regulation of downstream genes—*UCP1*.

### 4.4. Other Genes in Low Temperature Challenges

The inositol hexakisphosphate kinase-1 gene (*IP6K1*) was enriched in the phosphatidylinositol signaling system and up-regulated in all tissues of −5 °C Altay lambs compared to Hu lambs and −5 °C compared to the 20 °C Altay lambs, and down-regulated in the fat tissues of 20 °C Altay lambs compared to Hu lambs. However, the regulatory trends were reversed in the −5 °C compared to the 20 °C Hu lambs in fat tissues. In recent studies, *IP6K1* was reported to be related to adenosine monophosphate kinase (AMPK)-mediated thermogenesis [64] and that it regulates energy homeostasis through reducing AMPK activities in the adipose tissue browning process (certain WAT depots readily convert to a “brown-like” state with prolonged cold exposure or exposure to β-adrenergic compounds) and thermogenesis in adipose tissue [65]. Our results indicated that the *IP6K1* gene did not only differ between breeds, but also relieved the inhibition of AMPK-mediated UCP1-dependent thermogenesis by reducing gene expression. This provides further evidence that tail-fat of cold-exposed Hu lambs was involved in UCP1-dependent thermogenesis.

The *ADIPOQ* gene in the present study was down-regulated in the hypothalamus of −5 °C compared to 20 °C Altay lambs. It was reported that peripheral tissue ADIPOQ-deficient mice increased energy expenditure [66], which suggests cold exposure caused increased energy expenditure in Altay lambs. The *LPL* gene was up-regulated in the tail-fat of −5 °C Altay lambs compared to Hu lambs. The *LPL* gene can hydrolyze lipoproteins in BAT to provide free fatty acids for heat production and lead to an increase of serum TG concentration [23,24], and the *LPL* expression enhances fatty acid uptake from plasma triglycerides in WAT, at least in mice [67]. The browning marker gene (*UCP1*) was down-regulated in the tail-fat of −5 °C Altay lambs compared to Hu lambs but there was has no difference in the tail-fat of −5 °C compared to 20 °C Altay lambs, which suggests that this gene may not be involved in thermogenesis of BAT, but may play an important role in fat deposition in −5 °C Altay lambs compared to Hu lambs.

## 5. Conclusions

Previous studies on cold stress in sheep focused mainly on physiological responses [5,68,69]. We studied gene expression in key tissues involved in thermogenesis in lambs and expected to identify important thermogenic pathways and candidate thermogenic genes which play important roles in cold resistance in sheep. Based on the differentially expressed gene analysis in the present study and previous cold stress studies in other mammalian species, we concluded that there are differences in cold tolerance between Hu and Altay sheep. The hypothalamus plays an important role in responding to low temperature in the two breeds and is involved in heat production by the calcium signaling pathway in cold-exposed Altay lambs. The large difference in tail fat between Hu sheep and Altay sheep after cold exposure may be related to the different tail fat type in Altay (large fat tail) and Hu sheep (moderate fat tail). UCP1-dependent and other thermogenic pathways were activated when the lambs were exposed to cold.

## Figures and Tables

**Table 1:** Primer sequences for amplification of selected genes for RT-PCR quantification.

Gene Name	Primer Sequences (5′–3′)	Products Size (bp)	Tm (°C)
*UCP1*	ACTTCGTGTCCGCTGTTGTTG	104	61
	TGTGTACTGTCCTGGTGAAGAGTT		
*RYR1*	CAAGTGCTTCATCTGCGGTAT	83	60
	TGTGTTCCTCCAGTGTGTGAGT		
*ADIPOQ*	GAACAGTCCACAGGTCTAC	190	61
	CCTTCCATTACCACTACATAAC		
*LPL*	CGACAGGATTACAAGAGGAA	100	60
	AGGAATGAGGTGGCAAGT		
*β-actin*	AGCCTTCCTTCCTGGGCATGGA	113	60
	GGACAGCACCGTGTTGGCGTAGA		

## Data Availability

Data is contained within the article and Supplementary Material.

## Supplementary Materials

The following are available online at https://www.mdpi.com/2073-4425/12/3/375/s1, Supplementary File 1: Figure S1. Top 10 of up- and down-regulated DEGs enriched GO terms (CC and MF term) in different tissues and sheep breeds under cold exposure, Figure S2. Expression levels of four candidate genes from RT-PCR. Supplementary File S2: Table S1. Summary of transcriptome sequencing quality. Supplementary File S3: Table S2. Summary of DEGs in different compared groups. Supplementary File S4: Table S3. GO terms significantly enriched in three different tissues at different temperatures in the two breeds. Supplementary File S5: Table S4. KEGG pathways significantly enriched in three different tissues at different temperatures in the two breeds. Supplementary File S6: Table S5. Up- and down-regulated KEGG pathways significantly enriched in three different tissues at different temperatures in the two breeds. Supplementary File S7: Table S6. Effect of air temperature on feed intake and growth performance in Altay and Hu sheep; Table S7. Effect of air temperature on blood hormone and metabolite concentrations in Altay and Hu sheep.

## References

[1] Stott A.W., Slee J. (1985). The effect of environmental temperature during pregnancy on thermoregulation in the newborn lamb. Anim. Sci..

[2] Alexander G., Williams D. (1968). Shivering and non-shivering thermogenesis during summit metabolism in young lambs. J. Physiol..

[3] Clarke L., Heasman L., Firth K., E Symonds M. (1997). Influence of feeding and ambient temperature on thermoregulation in newborn lambs. Exp. Physiol..

[4] Kazak L., Chouchani E.T., Jedrychowski M.P., Erickson B.K., Shinoda K., Cohen P., Vetrivelan R., Lu G.Z., Laznik-Bogoslavski D., Hasenfuss S.C. (2015). A Creatine-Driven Substrate Cycle Enhances Energy Expenditure and Thermogenesis in Beige Fat. Cell.

[5] Sykes A.R., Griffiths R.G., Slee J. (1976). Influence of breed, birth weight and weather on the body temperature of newborn lambs. Anim. Sci..

[6] Slee J., Simpson S.P., Woolliams J.A. (1987). Metabolic rate responses to cold and to exogenous noradrenaline in newborn Scottish Blackface lambs genetically selected for high or low resistance to cold. Anim. Sci..

[7] Slee J., Stott A.W. (1986). Genetic selection for cold resistance in Scottish Blackface lambs. Anim. Sci..

[8] Li Y., Li Y.B., Liu C.J. (2017). Changes in lipid oxidation and fatty acids in Altay sheep fat during a long time of low temperature storage. J. Oleo Sci..

[9] Li J., Zhu X., Ma L., Xu H., Cao X., Luo R., Chen H., Sun X., Cai Y., Lan X. (2017). Detection of a new 20-bp insertion/deletion (indel) within sheep PRND gene using mathematical expectation (ME) method. Prion.

[10] Chen Y., Chen Y., Shi C., Huang Z., Zhang Y., Li S., Li Y., Ye J., Yu C., Li Z. (2018). SOAPnuke: A MapReduce acceleration-supported software for integrated quality control and preprocessing of high-throughput sequencing data. Gigascience.

[11] Kim D., Langmead B., Salzberg S.L. (2015). HISAT: A fast spliced aligner with low memory requirements. Nat. Methods.

[12] Langmead B., Salzberg S.L. (2012). Fast gapped-read alignment with Bowtie 2. Nat. Methods.

[13] Li B., Dewey C.N. (2011). RSEM: Accurate transcript quantification from RNA-Seq data with or without a reference genome. BMC Bioinform..

[14] Buja A., Eyuboglu N. (1992). Remarks on Parallel Analysis. Multivar. Behav. Res..

[15] Linting M., Van Os B.J., Meulman J.J. (2011). Statistical Significance of the Contribution of Variables to the PCA solution: An Alternative Permutation Strategy. Psychometrika.

[16] Wang L., Feng Z., Wang X., Wang X., Zhang X. (2009). DEGseq: An R package for identifying differentially expressed genes from RNA-seq data. Bioinformatics.

[17] Benjamini Y., Hochberg Y. (1995). Controlling the False Discovery Rate—A Practical and Powerful Approach to Multiple Testing. J. R. Stat. Soc. Ser. B-Methodol..

[18] Ashburner M., Ball C.A., Blake J.A., Botstein D., Butler H., Cherry J.M., Davis A.P., Dolinski K., Dwight S.S., Eppig J.T. (2000). Gene Ontology: Tool for the unification of biology. Nat. Genet..

[19] Ogata H., Goto S., Sato K., Fujibuchi W., Bono H., Kanehisa M. (1999). KEGG: Kyoto Encyclopedia of Genes and Genomes. Nucleic Acids Res..

[20] Livak K.J., Schmittgen T.D. (2001). Analysis of relative gene expression data using real-time quantitative PCR and the 2^−ΔΔCT^ Method. Methods.

[21] Shannon P., Markiel A., Ozier O., Baliga N.S., Wang J.T., Ramage D., Amin N., Schwikowski B., Ideker T. (2003). Cytoscape: A Software Environment for Integrated Models of Biomolecular Interaction Networks. Genome Res..

[22] Qiao L., Yoo H.S., Bosco C., Lee B., Feng G.-S., Schaack J., Chi N.-W., Shao J. (2014). Adiponectin reduces thermogenesis by inhibiting brown adipose tissue activation in mice. Diabetologia.

[23] Laperrousaz E., Denis R.G., Kassis N., Contreras C., López M., Luquet S., Cruciani-Guglielmacci C., Magnan C. (2018). Lipoprotein Lipase Expression in Hypothalamus Is Involved in the Central Regulation of Thermogenesis and the Response to Cold Exposure. Front. Endocrinol..

[24] Wang H., Eckel R.H. (2009). Lipoprotein lipase: From gene to obesity. Am. J. Physiol. Metab..

[25] Oliver S.R., Anderson K.J., Hunstiger M.M., Andrews M.T. (2019). Turning down the heat: Down-regulation of sarcolipin in a hibernating mammal. Neurosci. Lett..

[26] Kashan N., Azar G.M., Afzalzadeh A., Salehi A. (2005). Growth performance and carcass quality of fattening lambs from fat-tailed and tailed sheep breeds. Small Rumin. Res..

[27] Attia N., Bocquierb F., Khaldi G. (2004). Performance of the fat-tailed Barbarine sheep in its environment: Adaptive capacity to alternation of underfeeding and re-feeding periods. A review. Anim. Res..

[28] Gan S.Z.W., Shen M., Liang Y., Yang J., Gao L., Liu S., Wang X. (2013). Poly-morphism detection and analysis of the position (59327581) on X chromosome among sheep breeds of three different tail types. J. Shihezi Univ. (Nat. Sci.).

[29] Liu X., Li Q., Lin Q., Sun R. (2001). Uncoupling protein1 mRNA, mitochondrial GTP-binding, and T4 5′-deiodinase of brown adipose tissue in euthermic Daurian ground squirrel during cold exposure. Comp. Biochem. Physiol. Part A Mol. Integr. Physiol..

[30] Bieber L.L., Pettersson B., Lindberg O. (1975). Studies on Norepinephrine-Induced Efflux of Free Fatty Acid from Hamster Brown-Adipose-Tissue Cells. JBIC J. Biol. Inorg. Chem..

[31] Bukowiecki L., Folléa N., Lupien J., Paradis A. (1981). Metabolic relationships between lipolysis and respiration in rat brown adipocytes. The role of long chain fatty acids as regulators of mitochondrial respiration and feedback inhibitors of lipolysis. J. Biol. Chem..

[32] Zhou J., Ji K., Liu H., Zhang Y., Degen A.A., Jiao D., Wang W., Xie Z., Wang X., Zhou P. (2020). Effect of air temperature on growth performance, apparent digestibilities, rumen fermentation and serum metabolites in Altay and Hu lambs. J. Anim. Physiol. Anim. Nutr..

[33] Sano H., Sawada H., Takenami A., Oda S., Al-Mamun M. (2007). Effects of dietary energy intake and cold exposure on kinetics of plasma glucose metabolism in sheep. J. Anim. Physiol. Anim. Nutr..

[34] Stefanidis A., Wiedmann N.M., Tyagi S., Allen A.M., Watt M.J., Oldfield B.J. (2018). Insights into the neurochemical signature of the Innervation of Beige Fat. Mol. Metab..

[35] Martinez-Demena R., Anedda A., Cadenas S., Obregon M.-J. (2015). TSH effects on thermogenesis in rat brown adipocytes. Mol. Cell. Endocrinol..

[36] Cannon B., Houstek J., Nedergaard J. (1998). Brown Adipose Tissue: More Than an Effector of Thermogenesis?. Ann. N. Y. Acad. Sci..

[37] Daval M., Foufelle F., Ferré P. (2006). Functions of AMP-activated protein kinase in adipose tissue. J. Physiol..

[38] Chu S., Narayan V.P., Sung M.-K., Park T. (2017). Piperonal attenuates visceral adiposity in mice fed a high-fat diet: Potential involvement of the adenylate cyclase-protein kinase A dependent pathway. Mol. Nutr. Food Res..

[39] Tong T., Shen Y., Lee H.-W., Yu R., Park T. (2016). Adenylyl cyclase 3 haploinsufficiency confers susceptibility to diet-induced obesity and insulin resistance in mice. Sci. Rep..

[40] Vansal S.S. (2004). β3-Adrenergic Receptor Agonists and Other Potential Anti-obesity Agents. Am. J. Pharm. Educ..

[41] Marhl M., Haberichter T., Brumen M., Heinrich R. (2000). Complex calcium oscillations and the role of mitochondria and cytosolic proteins. Biosystems.

[42] Grubelnik V., Larsen A.Z., Kummer U., Olsen L.F., Marhl M. (2001). Mitochondria regulate the amplitude of simple and complex calcium oscillations. Biophys. Chem..

[43] Hu R., He M.-L., Hu H., Yuan B.-X., Zang W.-J., Lau C.-P., Tse H.-F., Li G.-R. (2009). Characterization of calcium signaling pathways in human preadipocytes. J. Cell. Physiol..

[44] Andrienko T., Kuznetsov A.V., Kaambre T., Usson Y., Orosco A., Appaix F., Tiivel T., Sikk P., Vendelin M., Margreiter R. (2003). Metabolic consequences of functional complexes of mitochondria, myofibrils and sarcoplasmic reticulum in muscle cells. J. Exp. Biol..

[45] Shkryl V.M., Shirokova N. (2006). Transfer and Tunneling of Ca^2+^ from Sarcoplasmic Reticulum to Mitochondria in Skeletal Muscle. J. Biol. Chem..

[46] De Meis L., Oliveira G.M., Arruda A.P., Santos R., Da Costa R.M., Benchimol M. (2005). The Thermogenic Activity of Rat Brown Adipose Tissue and Rabbit White Muscle Ca^2+^-ATPase. IUBMB Life.

[47] Gnad T., Scheibler S., Von Kügelgen I., Scheele C., Kilić A., Glöde A., Hoffmann L.S., Reverte-Salisa L., Horn P., Mutlu S. (2014). Adenosine activates brown adipose tissue and recruits beige adipocytes via A2A receptors. Nat. Cell Biol..

[48] Leaver E.V., Pappone P.A. (2002). β-Adrenergic potentiation of endoplasmic reticulum Ca^2+^ release in brown fat cells. Am. J. Physiol. Physiol..

[49] Strosberg A.D. (1997). STRUCTURE AND FUNCTION OF THE β3-ADRENERGIC RECEPTOR. Annu. Rev. Pharmacol. Toxicol..

[50] Henry B.A., Pope M., Birtwistle M., Loughnan R., Alagal R., Fuller-Jackson J.-P., Perry V., Budge H., Clarke I.J., Symonds M.E. (2017). Ontogeny and Thermogenic Role for Sternal Fat in Female Sheep. Endocrinology.

[51] Hampton M., Melvin R.G., Andrews M.T. (2013). Transcriptomic Analysis of Brown Adipose Tissue across the Physiological Extremes of Natural Hibernation. PLoS ONE.

[52] Orozco-Solis R., Aguilar-Arnal L., Murakami M., Peruquetti R., Ramadori G., Coppari R., Sassone-Corsi P. (2016). The Circadian Clock in the Ventromedial Hypothalamus Controls Cyclic Energy Expenditure. Cell Metab..

[53] Kurylowicz A., Jonas M., Lisik W., Jonas M., Wicik Z.A., Wierzbicki Z., Chmura A., Puzianowska-Kuznicka M. (2015). Obesity is associated with a decrease in expression but not with the hypermethylation of thermogenesis-related genes in adipose tissues. J. Transl. Med..

[54] De Meis L. (2003). Brown Adipose Tissue Ca^2+^-ATPase: Uncoupled ATP hydrolysis and thermogenic activity. J. Biol. Chem..

[55] De Meis L., Arruda A.P., Da Costa R.M., Benchimol M. (2006). Identification of a Ca^2+^-ATPase in Brown Adipose Tissue Mitochondria: Regulation of thermogenesis by ATP and Ca^2+^. J. Biol. Chem..

[56] Bal N.C., Sahoo S.K., Maurya S.K., Periasamy M. (2018). The Role of Sarcolipin in Muscle Non-shivering Thermogenesis. Front. Physiol..

[57] Bal N.C., Singh S., Reis F.C.G., Maurya S.K., Pani S., Rowland L.A., Periasamy M. (2017). Both brown adipose tissue and skeletal muscle thermogenesis processes are activated during mild to severe cold adaptation in mice. J. Biol. Chem..

[58] Yao C.-K., Liu Y.-T., Lee I.-C., Wang Y.-T., Wu P.-Y. (2017). A Ca^2+^ channel differentially regulates Clathrin-mediated and activity-dependent bulk endocytosis. PLoS Biol..

[59] El-Merahbi R., Löffler M., Mayer A., Sumara G. (2015). The roles of peripheral serotonin in metabolic homeostasis. FEBS Lett..

[60] Sumara G., Sumara O., Kim J.K., Karsenty G. (2012). Gut-Derived Serotonin Is a Multifunctional Determinant to Fasting Adaptation. Cell Metab..

[61] Söhle J., Machuy N., Smailbegovic E., Holtzmann U., Grönniger E., Wenck H., Stäb F., Winnefeld M. (2012). Identification of New Genes Involved in Human Adipogenesis and Fat Storage. PLoS ONE.

[62] Harms M., Seale P. (2013). Brown and beige fat: Development, function and therapeutic potential. Nat. Med..

[63] Bostroem P., Wu J., Jedrychowski M.P., Korde A., Ye L., Lo J.C., Rasbach K.A., Bostroem E.A., Choi J.H., Long J.Z. (2012). A PGC1-α-dependent myokine that drives brown-fat-like development of white fat and thermogenesis. Nat. Cell Biol..

[64] Zhu Q., Ghoshal S., Rodrigues A., Gao S., Asterian A., Kamenecka T.M., Barrow J.C., Chakraborty A. (2016). Adipocyte-specific deletion of Ip6k1 reduces diet-induced obesity by enhancing AMPK-mediated thermogenesis. J. Clin. Investig..

[65] Zhu Q., Ghoshal S., Tyagi R., Chakraborty A. (2017). Global IP6K1 deletion enhances temperature modulated energy expenditure which reduces carbohydrate and fat induced weight gain. Mol. Metab..

[66] Kubota N., Yano W., Kubota T., Yamauchi T., Itoh S., Kumagai H., Kozono H., Takamoto I., Okamoto S., Shiuchi T. (2007). Adiponectin Stimulates AMP-Activated Protein Kinase in the Hypothalamus and Increases Food Intake. Cell Metab..

[67] Duivenvoorden I., Teusink B., Rensen P.C., Romijn J.A., Havekes L.M., Voshol P.J. (2005). Apolipoprotein C3 Deficiency Results in Diet-Induced Obesity and Aggravated Insulin Resistance in Mice. Diabetes.

[68] Sahoo A., Paul R.K., Thirumurgan P., Sharma S., Kumawat P.K., De K. (2019). Immunological and plasma antioxidant re-sponse following protection of newborn lambs from cold by umbrella-type housing and lamb-jacket in winter. Biol. Rhythm Res..

[69] Sejian V., Kumar D., Naqvi S.M.K. (2017). Physiological rhythmicity in Malpura ewes to adapt to cold stress in a semi-arid tropical environment. Biol. Rhythm Res..

