# Supplementary material for: Transcriptome Analysis Reveals Genes Involved in Thermogenesis in Two Cold-Exposed Sheep Breeds
Source: Genes (Basel). 2021 Mar 6;12(3):375. doi: 10.3390/genes12030375 (PMC7999592; doi:10.3390/genes12030375)
Supplement: Supplementary file 1 [file genes-12-00375-s001.zip › Additional files/Supplementary File S1.docx]

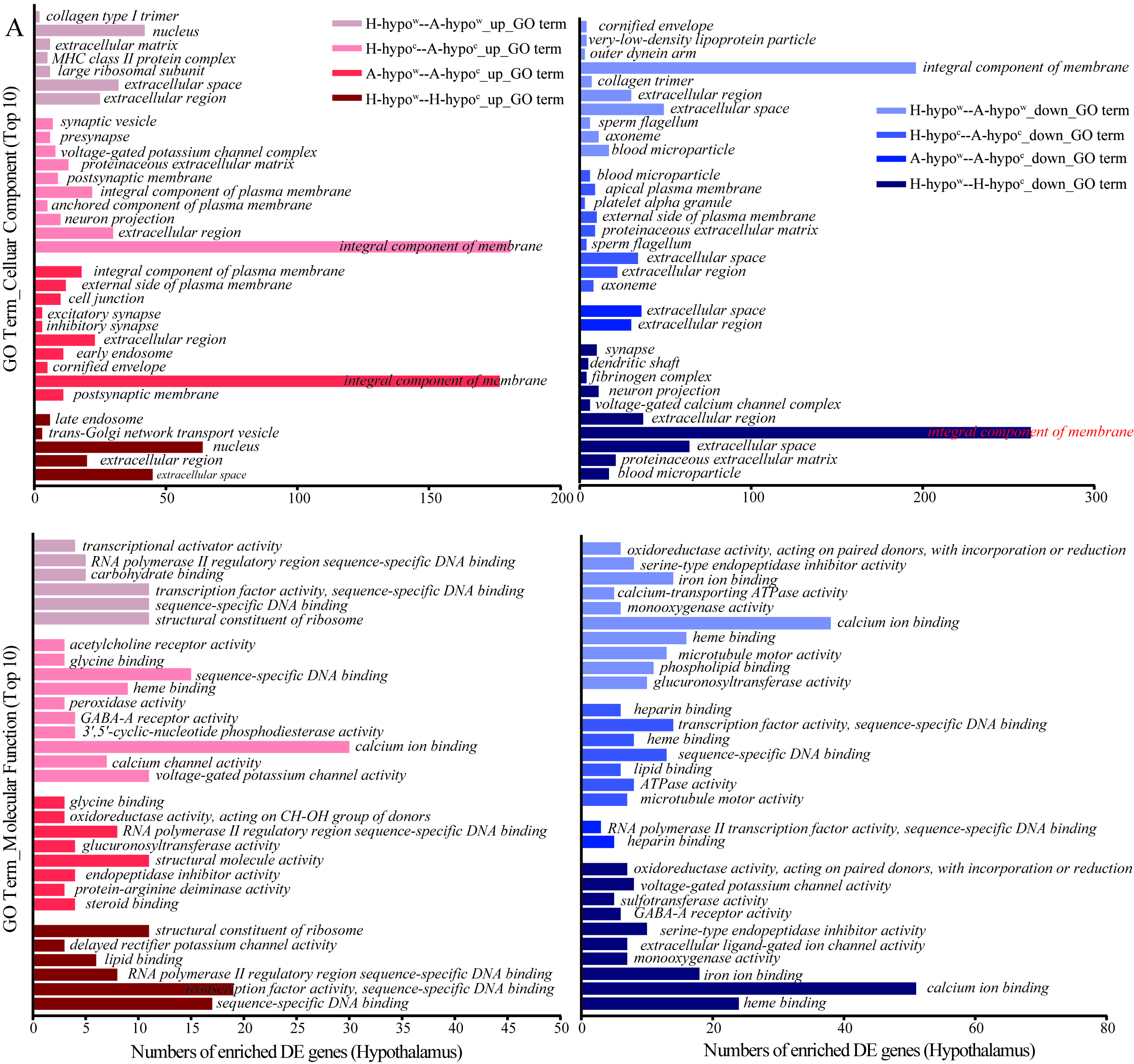


**(A)**

**Figure S1.** Top 10 of up- and down-regulated DEGs enriched GO terms (CC and MF terms) in different tissues and sheep breeds under cold exposure. (**A**): GO terms enriched in the hypothalamus, (**B**): GO terms enriched in the perirenal fat tissue, (**C**): GO terms enriched in the tail-fat tissue. A-hypo^c^: hypothalamus tissue of ^-^5 °C Altay lambs; A-hypo^w^: hypothalamus tissue of 20 °C Altay lambs; H-hypo^c^: hypothalamus tissue of ^-^5 °C Altay lambs; H-hypo^w^: hypothalamus tissue of 20 °C Hu lambs. A-peri^c^: perirenal fat tissue of ^-^5 °C Altay lambs; A-peri^w^: perirenal fat tissue of 20 °C Altay; H-peri^c^: perirenal fat tissue of ^-^5 °C Altay lambs; H-peri^w^: perirenal fat tissue of 20 °C Hu lambs. A-tail^c^: tail-fat tissue of ^-^5 °C Altay lambs; A-tail^w^: tail-fat tissue of 20 °C Altay lambs; H-tail^c^: tail-fat tissue of ^-^5 °C Altay lambs; H-tail^w^: tail-fat tissue of 20 °C Hu lambs. (P < 0.05). Red represented up-regulated GO terms and represented down-regulated GO terms.


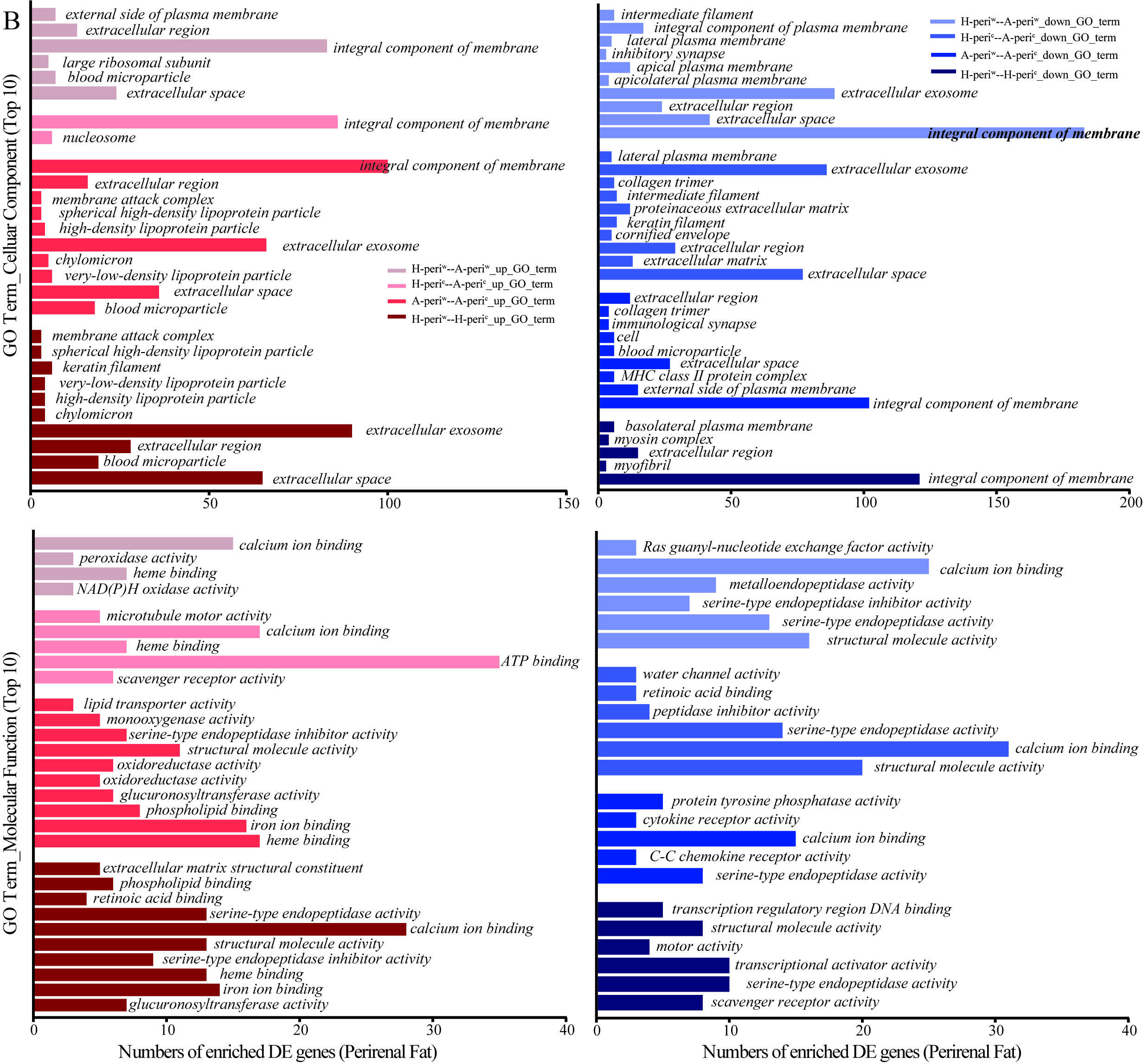


**(B)**

**Figure S1.** Top 10 of up- and down-regulated DEGs enriched GO terms (CC and MF terms) in different tissues and sheep breeds under cold exposure. (**A**): GO terms enriched in the hypothalamus, (**B**): GO terms enriched in the perirenal fat tissue, (**C**): GO terms enriched in the tail-fat tissue. A-hypo^c^: hypothalamus tissue of ^-^5 °C Altay lambs; A-hypo^w^: hypothalamus tissue of 20 °C Altay lambs; H-hypo^c^: hypothalamus tissue of ^-^5 °C Altay lambs; H-hypo^w^: hypothalamus tissue of 20 °C Hu lambs. A-peri^c^: perirenal fat tissue of ^-^5 °C Altay lambs; A-peri^w^: perirenal fat tissue of 20 °C Altay; H-peri^c^: perirenal fat tissue of ^-^5 °C Altay lambs; H-peri^w^: perirenal fat tissue of 20 °C Hu lambs. A-tail^c^: tail-fat tissue of ^-^5 °C Altay lambs; A-tail^w^: tail-fat tissue of 20 °C Altay lambs; H-tail^c^: tail-fat tissue of ^-^5 °C Altay lambs; H-tail^w^: tail-fat tissue of 20 °C Hu lambs. (P < 0.05). Red represented up-regulated GO terms and represented down-regulated GO terms.


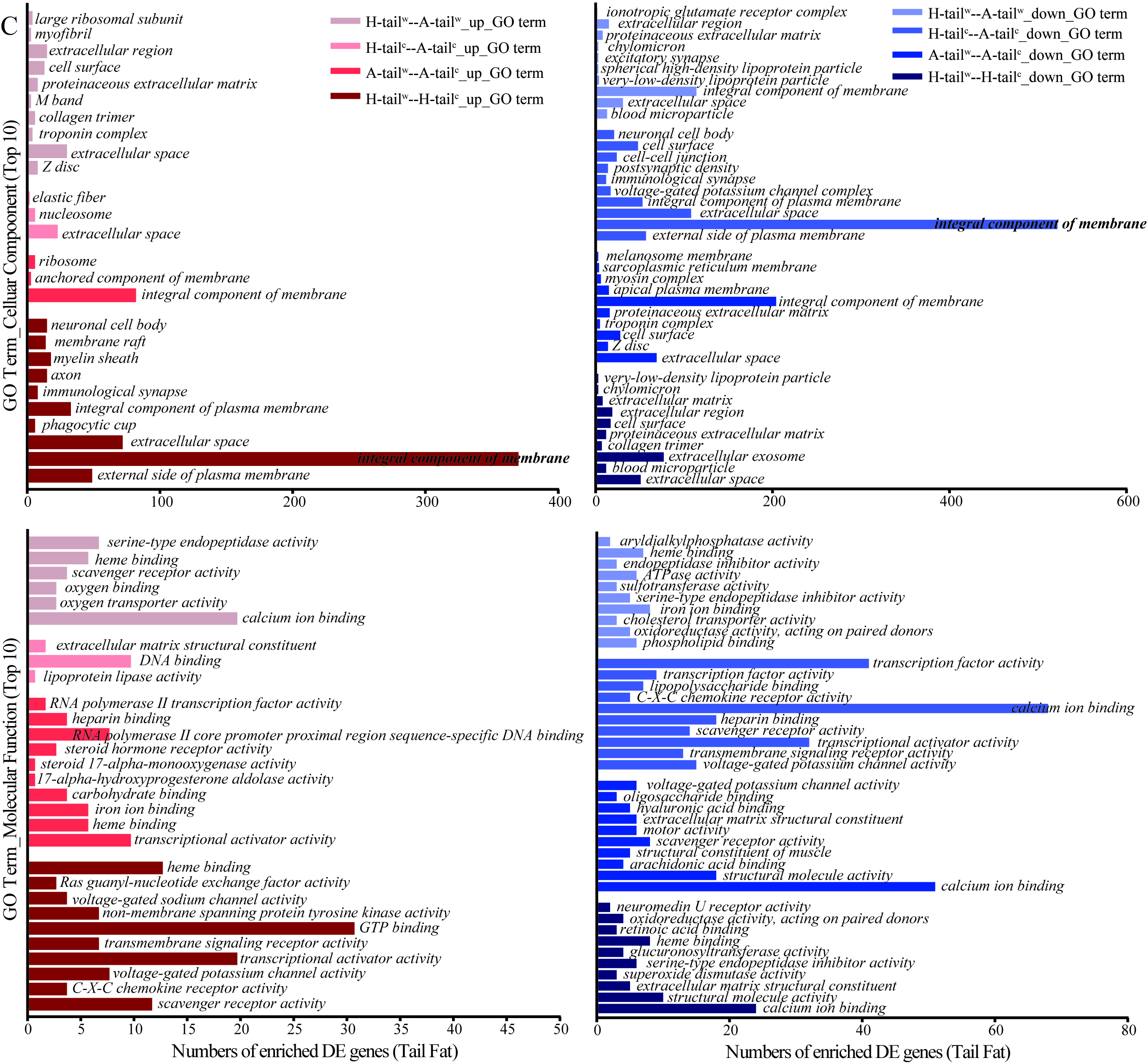


**(C)**

**Figure S1.** Top 10 of up- and down-regulated DEGs enriched GO terms (CC and MF terms) in different tissues and sheep breeds under cold exposure. (**A**): GO terms enriched in the hypothalamus, (**B**): GO terms enriched in the perirenal fat tissue, (**C**): GO terms enriched in the tail-fat tissue. A-hypo^c^: hypothalamus tissue of ^-^5 °C Altay lambs; A-hypo^w^: hypothalamus tissue of 20 °C Altay lambs; H-hypo^c^: hypothalamus tissue of ^-^5 °C Altay lambs; H-hypo^w^: hypothalamus tissue of 20 °C Hu lambs. A-peri^c^: perirenal fat tissue of ^-^5 °C Altay lambs; A-peri^w^: perirenal fat tissue of 20 °C Altay; H-peri^c^: perirenal fat tissue of ^-^5 °C Altay lambs; H-peri^w^: perirenal fat tissue of 20 °C Hu lambs. A-tail^c^: tail-fat tissue of ^-^5 °C Altay lambs; A-tail^w^: tail-fat tissue of 20 °C Altay lambs; H-tail^c^: tail-fat tissue of ^-^5 °C Altay lambs; H-tail^w^: tail-fat tissue of 20 °C Hu lambs. (P < 0.05). Red represented up-regulated GO terms and represented down-regulated GO terms.


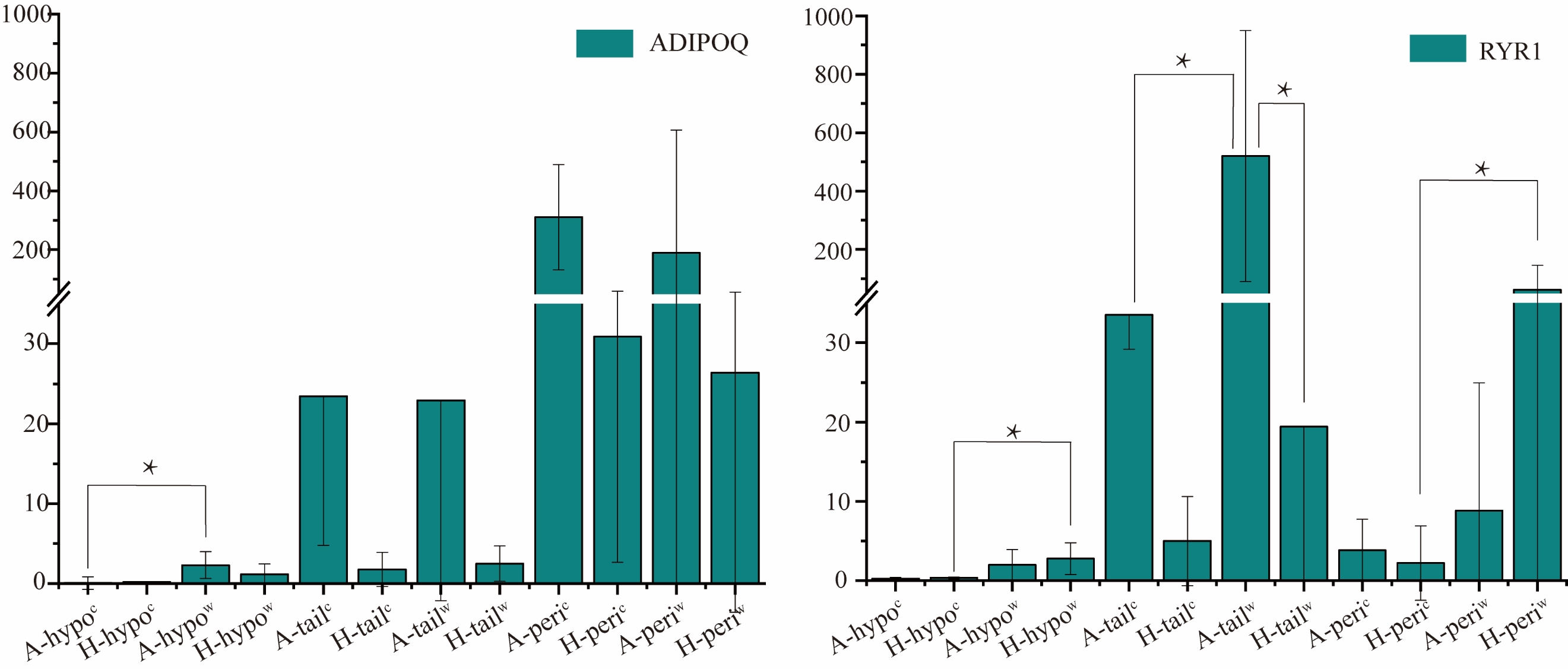


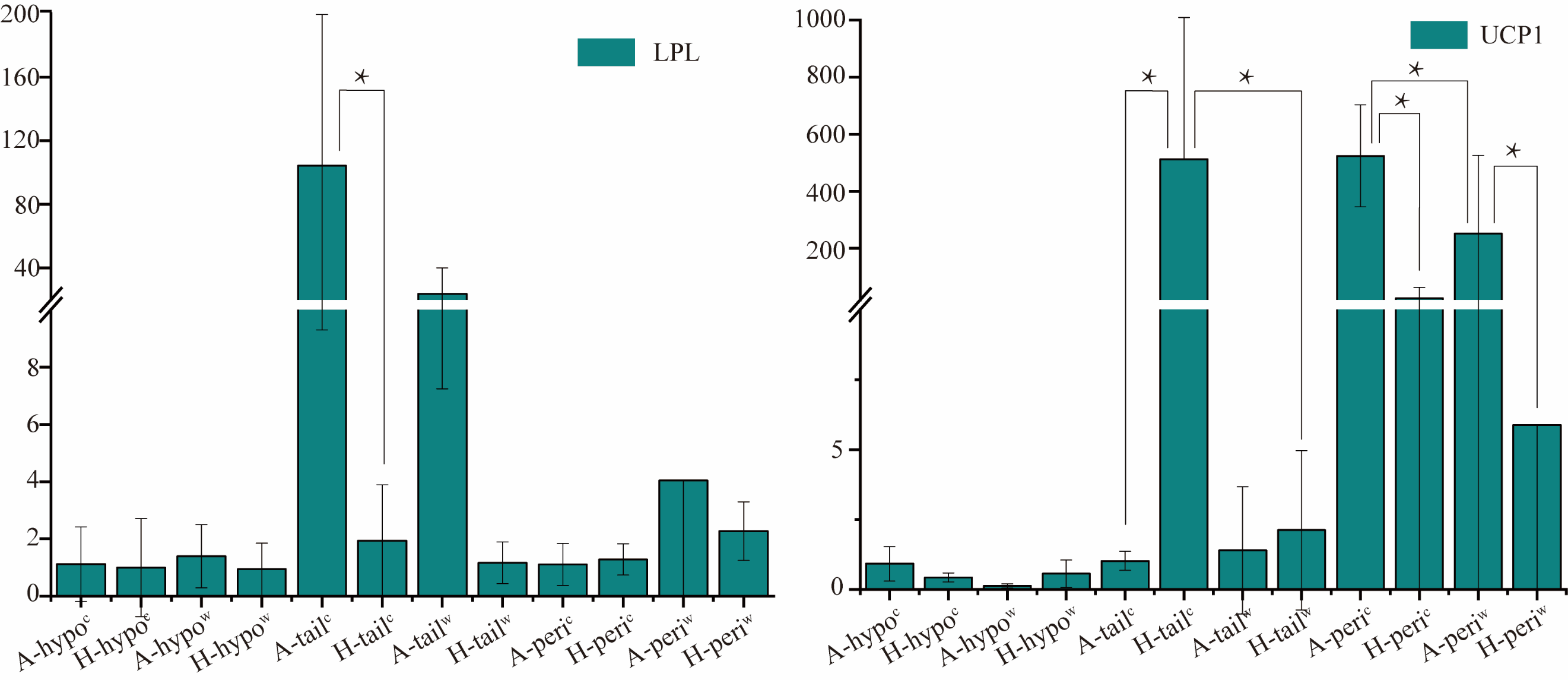


**Figure S2.** Expression levels of four candidate genes from RT-PCR. The X-axis represents the different adipose tissues; the Y-axis represents the relative gene expression levels of RT-PCR using columns and bars. Significant differences are represented by * (*p* < 0.05).
