# Supplementary material for: Transcriptome Analysis Reveals Genes Involved in Thermogenesis in Two Cold-Exposed Sheep Breeds
Source: Genes (Basel). 2021 Mar 6;12(3):375. doi: 10.3390/genes12030375 (PMC7999592; doi:10.3390/genes12030375)
Supplement: Supplementary file 1 [file genes-12-00375-s001.zip › Additional files/Supplementary File S7.docx]

**Table S6.** Effect of air temperature on feed intakes and growth performance in Altay and in Hu sheep.

| **Items** | **−5 °C** | |  | **20 °C** | |  | ***p*-value** | | |
| --- | --- | --- | --- | --- | --- | --- | --- | --- | --- |
|  | **Altay** | **Hu** |  | **Altay** | **Hu** | **SEM** | **Breed** | **Temperature** | **interaction** |
| DM intake, g/d DM | 1991 | 1753 |  | 1702 | 1409 | 69.4 | <0.01 | <0.001 | 0.607 |
| DM intake, g kg^-0.75^d^-1^ | 147 | 127 |  | 133 | 109 | 4.2 | 0.017 | <0.001 | 0.305 |
| ME intake, MJ/d | 15.7 | 14.6 |  | 13.8 | 11.2 | 0.58 | <0.01 | <0.01 | 0.169 |
| ME intake, MJ kg^-0.75^d^-1^ | 1.82 | 1.58 |  | 1.65 | 1.35 | 0.053 | 0.017 | <0.001 | 0.305 |
| Average daily gain, g/d | 204 | 118 |  | 156 | 100 | 9.80 | <0.001 | <0.01 | 0.144 |
| Body mass gain: DM intake (g/g) | 0.103 | 0.067 |  | 0.091 | 0.071 | 0.0048 | <0.001 | 0.466 | 0.154 |

DM, dry matter; ME, metabolizable energy.

**Table S7.** Effect of air temperature on blood hormone and metabolite concentrations in Altay and Hu sheep.

| **Items** | **−5 °C** | |  | **20 °C** | |  | ***p*-value** | | |
| --- | --- | --- | --- | --- | --- | --- | --- | --- | --- |
|  | **Altay** | **Hu** |  | **Altay** | **Hu** | **SEM** | **Breed** | **Temperature** | **Interaction** |
| Glucose, mmol/L | 4.38 | 5.87 |  | 4.27 | 5.72 | 0.273 | <0.001 | 0.641 | 0.952 |
| NEFA, mmol/L | 0.42 | 0.58 |  | 0.46 | 0.58 | 0.046 | 0.012 | 0.665 | 0.693 |
| BHBA, mmol/L | 0.25 | 0.27 |  | 0.23 | 0.27 | 0.013 | 0.061 | 0.366 | 0.296 |
| Urea-N, mmol/L | 9.21 | 10.52 |  | 9.53 | 10.56 | 0.358 | 0.030 | 0.405 | 0.518 |
| GH, ng/mL | 6.99 | 7.14 |  | 7.74 | 8.67 | 0.605 | 0.395 | 0.090 | 0.530 |
| Insulin, μIU/mL | 26.7 | 26.7 |  | 27.6 | 28.1 | 1.07 | 0.798 | 0.344 | 0.833 |
| IGF-1, ng/mL | 257 | 253 |  | 251 | 258 | 18.1 | 0.950 | 0.975 | 0.746 |
| Glucagon, pg/mL | 150 | 155 |  | 153 | 159 | 22.9 | 0.821 | 0.871 | 0.959 |
| Cortisol, ng/mL | 11.22 | 10.72 |  | 10.70 | 10.96 | 0.963 | 0.929 | 0.746 | 0.402 |
| T3, nmol/L | 3.69 | 4.00 |  | 4.27 | 4.20 | 0.425 | 0.836 | 0.150 | 0.464 |
| T4, nmol/L | 39.1 | 44.7 |  | 44.7 | 40.1 | 5.41 | 0.935 | 0.923 | 0.343 |

NEFA, nonesterified fatty acid; BHBA, β-hydroxybutyrate; GH, growth hormone; IGF-1, insulin-like growth factor-1; T3, triiodothyonine; T4, thyroxine.
